# Issues of therapeutic communication relevant for improving quality of care

**Published:** 2014

**Authors:** O Popa-Velea, VL Purcărea

**Affiliations:** * Department of Medical Psychology, University of Medicine and Pharmacy “Carol Davila”, Bucharest; ** Department of Marketing, University of Medicine and Pharmacy “Carol Davila”, Bucharest

**Keywords:** communication, patient, doctor, quality of care

## Abstract

Communication issues are extensively considered a topic of high interest for improving the efficacy of the therapeutic act. This article aimed to overview several issues of therapeutic communication relevant for improving quality of care. A number of 15 bibliographic resources on these topics published in peer-reviewed journals between 1975 and 2010, and indexed in PubMed, ProQuest and EBSCO databases were examined, to seek for evidence regarding these data. Results highlight a number of communication problems commonly reported in the literature, such as the lack of physician communicational skills or their deterioration, the persistence of an asymmetric therapeutic communicational model, communication obstacles brought by the disease itself or by several variables pertaining to the patient, including specific demographic and psychological contexts. Equally, literature reports ways of improving therapeutic communication, such as optimizing the clinical interview, better time management techniques or assertiveness. Integration of communication training in the bio-psycho-social model of care and monitoring parameters like adherence and quality of life as tools reflecting also a good therapeutic communication can be valuable future approaches of obtaining better results in this area.

## Introduction

Communication issues are extensively considered a matter of interest, both for clinicians and researchers, especially since, in the last decades, patients have become increasingly informed about their conditions, and involved in the decision-making processes [**1**, **2**]. In those medical specialties where “the emotional dimension is omnipresent” (**3**) and the probability of distress and psychological symptoms is often high, communication gets a particular importance, throughout the whole care pathway. 

Improving communication can lead to a number of palpable benefits [**4**-**6**]:

- it facilitates description of symptoms, thereby increasing diagnosis accuracy, and a better understanding of medical indications;

- offers a reliable tool for detecting the emotional states of the patient and for orienting him/her towards the most adequate therapeutic measures; 

- offers the chance of identifying patient’s needs, perceptions or apprehensions regarding the disease and the treatment;

- offers the premise for including the patient in taking medical decisions

The communication abilities of the physician are generally assessed by patients as being important or very important, being perceived by many of them as comparable, or even occasionally more significant than technical competence [**7**]. 

An important component of patient’s psychological comfort is represented by the control over the symptom, which is proportionally to the ability to perceive the information received from the physician as being meaningful or accurate. Last but not least, the quality of the doctor-patient communication has a direct influence on adherence, especially in chronic conditions, where the differences brought by various treatments and caring persons are accounted for merely by the communicational level, than by the efficiency of a certain therapeutic regimen.

## Aim, method

This paper overviews several issues of therapeutic communication relevant for improving quality of care. A number of 15 bibliographic resources on these topics (marked with * in the reference list), published in peer-reviewed journals between 1975 and 2010, and indexed in PubMed, ProQuest and EBSCO databases were examined, to seek for evidence regarding these data. 

## Results

Our results are grouped around (3.1.) the main types of problems reported in therapeutic relationships and (3.2.) the solutions to overcome or prevent them.

**Problems**

Communication problems are reported, conceptualized and reported typically in a different manner by the physician and the patient. Being aware of both points of view improves the chance to adopt adequate measures to improve communication efficiency.

Most patients complain about two major communication flaws:

- *lack of physician communicational skills or their deterioration throughout the disease:* this is typically noticed more often at more experienced phyisicans, even if one would expect their previous therapeutic experiences enables them with higher willingness and ability to communicate. In practice, this is not always the case. Some authors consider this phenomenon could be due to the increasing technical competence of more experienced physicians, who do not invest, oppositely to their younger colleagues, in communicational skills, as they do not prove so much useful anymore to fulfill the healer role. Other hypothesis is related to circumstances such as empathy fatigue, rust-out and burnout syndrome, which tend to occur as the physician goes further in a demanding and stressful career. It is noteworthy that “holistic care” is quite rarely an objective of programs dedicated to continuous medical education [**8**]. Within most medical training programs, acquiring technical competence is still much more valued than learning relational skills, thereby empathy being implicitely marginalized and / or discouraged.

- *the persistence of an asymmetric therapeutic communicational model*, which precludes *ab initio *patient’s ability to understand key informations about the dynamics of its suffering or about the role of the medication. This leads to inadequate delivery of information to the patient or his/her relatives, schematic communicational rituals in the daily therapeutic encounter and, all in all, a perception of the physician as being emotionally incompetent or indifferent. In chronic and incurable diseases, such perceptions can influence significantly the global psychological status of the patient and quite often, the prognosis, expressed as life expectancy [**9**]. 

The assymetrical model of care has been proven to have negative consequences on the medical performance, leading quite often to:

- *the tendency to judge / criticize / label*, a key impediment in the development of a positive transference and mutual therapeutical trust; 

- *sanctionatory opinions and behaviors*, if the patient does not answer according to the rigid scenario of the perfect patient (one who is always motivated, willing to act in his/her own benefit, unceasingly ready to follow the received prescriptions, etc.);

- *avoidance, as an easy way to handle communication problems*. It is more frequent in those physicians for whom listening the patient is solely an obligatory phase to check, and not a (redoubtable) tool to get to know the complexity of the human being in need of help and support. In a system where the physician is perceived as the key or the only administrator of care, avoidance is met more often than expected, as it represents a way for the physician to protect himself from getting overwhelmed by “collateral problems” beside pure clinical work. In fact, namely because the physician is perceived by the patient as a key figure, avoidance is not the best way to handle difficult cases, because this typically leads to lack of trust, poor adherence and eventually to a bad prognosis, later impacting, in a boomerang-like effect, on the avoidant physician himself [**10**];

- *neglecting socio-cultural differences or approaching them in an adequate way:* it was proven to influence substantially the way some patients understand and accept their suffering. Theory of Reasoned Action [**11**, **12**] argues that norms and social motives play a key role in shaping attitudes towards the disease and behaviors related to them. For example, the patients with a low socio-economic status tend to use the medical institution more for healing than for prevention, to rely more on their entourage and sometimes to have unrealistic expectations from the physician. 

At their turn, physicians identify several other reasons for therapeutic communication difficulties:

- *the particularities of the disease itself: for example, in many psychological or psychiatric diseases*, doctors have to handle cognitive obstacles (such as the distorsion of messages which are exchanged inside the therapeutic relationship); in *selected somatic conditions*, a series of symptoms can restrain patient’s ability to explain, listen or understand (e.g. pain, neurologic disorders, etc.).

- *the characteristics of the patient:* personality type, for example, can occasionally offer a very rigid or limited framework for the doctor-patient relationship. Patients with personality disorders are often not aware of their inadequacy and do not invest in communication, as they do not expect or want any change in their lifestyle. Projection, as defense mechanism, is common, they do not feel supported or understood and tend to react through non-adherence, latent or manifest hostility, or negative transference [**13**].

- *demographic variables, such as age* (for example, elderly are exposed to limited or distorted communication, via cognitive impairments or age-illness stereotype). A common outcome is the negative counter-transference of the physician, caught between the demands of the healer role and the challenges of a patient who “does not want to heal”;

- *the socio-cultural context*, which creates a high variability on the way patients express their suffering and on their focus [**14**]. For example, patients from individualistic societies tend to be more focused on the interpretation of verbal messages come from the doctor. Inversely, those stemming from a collectivistic background are more trained to listen to others’ opinions, pay more attention to nonverbal cues and also tend to use more often the nonverbal ways of expression [**15**].

Although many situations evoked above can be handled quite successfully by the physician (especially if they are early identified and/or a collaboration is carried on with a psychologist, social worker or anthropologist), the basic rule remains to *prevent* the possible disorders in the sphere of communication. Prevention has the advantage to maintain close to the optimum the potential of therapeutic intervention, because prevention is able to conserve or enhance trust. 

**Posibilities of improving the therapeutic relationship **

Several of the ways through which the therapeutic relationship can be improved are commonly mentioned in current and past literature in the field. 

**Optimizing the clinical interview**

The interview should offer the chance to the patient of perceiving support and empathy and of building emotional catharsis and trust. This can be done from the very beginning of the conversation, encouraging free expression, via open-ended questions and nonverbal positive cues. In a next phase, a more focused approach would allow going through the main complaints of the patient, however the doctor should not omit [**17**]: 

- *obtaining a description not only of physical, but also of psychological symptoms / the emotional impact of illness*;

- *integrating the current symptoms in the larger context of patient’s life* (e.g getting information regarding the possible impact on the disease of family dynamics, risk factors, or sick role);

- *investigating how the disease is perceived and mentally reconstructed, its impact on patient’s hopes, expectancies about therapy and trust*.

In what concerns the factual content of the interview, phrasing the questions should take into consideration several elements:

- to use precise questions, and avoid those that are confusing or excessive in complexity and/or length; 

- to refrain from questions that may suggest the answer;

- to maintain a degree of cautiousness in taking patient’s statements as granted (some of them may come with prefabricated diagnoses, emerging from their own interpretations);

- to avoid aloud, real-time interpretations of gathered data;

- to pay attention, in a balanced way, to all the symptoms and complaints of the patient;

- to avoid embarassing questions, for example those that are focused on technical aspects, which are inaccessible to the patient;

- to be empathic, to show tact and consideration to the patient, during the entire interview;

- to use active listening as a reliable tool to maintain the emotional connection to the patient. This can be done using various procedures, like encouraging, paraphrasing, reflecting, resuming, or using nonverbal language (e.g. gestures showing openness / approval). Typically, active listening has substantial benefits, such as facilitating emotional catharsis and building / maintaining trust inside the therapeutic relationship;

In the case of “difficult patients”, the elements mentioned above can be supplemented with others that can furthermore increase the efficiency of communication [**17**] (**Table 1**).

**Table 1:** Interview content to be considered when dealing with a patient suffering from a psychosomatic or psychological disorder (Popa-Velea et al., 2013)

*Element of the interview*	*Usefulness*
Making an inventory of the whole set of problems, not just of the current issue	Highlights problems and correlations not obvious to the patient, but relevant for his / her suffering or which may explain previous failures
Testing patient’s emotional involvement and motivation	Offers information about the amount of resources the patient has in coping with his / her illness
Obtaining detailed information regarding the conditions in which the symptoms occur	Allows comprehension of certain patterns, such as the role of conditioned responses in symptom’s development and suggests ways to interrupt them
Understanding patient’s vision on the illness and the treatment	Warns the therapist about possible issues which may increase resistance to therapy
Investigating the coping strategies that are the most preferred by the patient in problematic situations	Uncovers unproductive, harmful or inefficient coping strategies, possibly approachable via counseling or psychotherapy
Exploring the psychological impact of the treatment / of previous hospitalizations	Has a contribution in increasing the level of “basic” trust in the relationship with the current therapist
Investigating the intensity and the quality of social and familial relationships	Allows discovery of underlying pathogenic elements (e.g. personality disorders). Reveals key individuals, able to provide support under critical circumstances.

**A better use of time for the therapeutic relationship**

Various literature data show that, generally, a positive communication relationship is developed proportionally to the frequency of interaction. Many patients complain about the insufficient time spent with the doctor and of their real impossibility to entirely reveal their fears and to obtain all the necessary advice. Apparently, this problem is difficult to solve, as the doctor has a limited time for each patient, and extending it could be detrimental to another one. Still, a solution exists and it has to do with the qualitative tone of the doctor-patient interaction [**18**, **19**]. The density of the therapeutic relationship can be variable, according to patient’s needs or the stage of the disease, but if it allows a genuine emotional exchange, expression of support and elaboration of a mutually-agreed therapeutic strategy, there are substantial chances that satisfaction of patients will be higher and more durable [**20**]. 

**Assertiveness – premises of an efficient communication in the doctor-patient relationship**

In order to enhance the doctor-patient communication and to consolidate this relationship, with positive effects concerning the therapeutic compliance and the favorable evolution of the disease, a very useful concept is assertiveness. It represents “’the ability to honestly express opinions, emotions and personal attitudes, without fear and without denying others’ rights” [**21**]. Assertiveness involves both openness and receptivity, within the limits of empathy.

In the doctor-patient relationship, assertiveness – considered by some authors as a veritable “dialogue medicine” [**22**] – should be constantly used, because it is an excellent tool for understanding the patient and an important ingredient of trust; the latter, in turn, has a positive, sometimes decisive, influence on therapeutic compliance. In the absence of assertiveness, negative consequences may appear, both for the physician and the patient [**14**] (**Table 2**).

**Table 2:** Possible emotional reactions and behavioral manifestations generated by non-assertiveness in the doctor-patient relationship (Cioca & Popa-Velea, 2013)

*Emotional reactions*	*Behavioral manifestations*	**
**	*Patient*	*Doctor*
*Depression*	Passivity, learned helplessness, hypo- or noncompliance	Avoiding the patient and his/her family, incapacity to help, emotional exhaustion, burnout
*Frustration*	Blaming the doctor, negative emotional transference; self blaming	The feeling of being manipulated, avoiding the patient, negative countertransference
*Alienation*	Hypo- or noncompliance, from perceiving as meaningless the relationship with the doctor and, consequently, the therapy	Feelings of non authenticity, ambivalence or professional failure (± fear of being discovered)
*Anger*	Aggressiveness; latent or manifest	Aggressiveness (mostly latent), avoiding contact with the patient, referring him/her to another doctor without explanations or invoking ridiculous reasons
*Disappointment*	Negative transference, hypo- or noncompliance, originating in exaggerated expectations from the doctor (without testing first his/her resources or those of the therapy)	Negative countertransference, originating in exaggerated expectations from the patient (without testing first his/her availability and capability to follow the treatment)

Educating health professionals into cultivating assertiveness has a certain number of advantages [**23**]:

- it serves both patient’s and doctor’s needs: the former, to “tell his/her story” (cathartic effect), and the latter, to listen and accurately understand, in order to decide for a correct treatment;

- it maximizes patient’s ability to express his/her complaints and deepest emotional states;

- it reflects and respects the congruence between the psychological state of the patient and his/her physical experience of the disease;

- it increases the efficiency of doctor’s knowledge and expertise;

- it represents a mutual gain, leading to a match of doctor’s and patient’s expectations;

- it helps participants overcome their stereotypical roles, so that both participants have the feeling of power and the freedom to change the context of their interaction;

- it facilitates the development of a solid doctor-patient relationship, and – subjacently – the dynamic and the results of medical care.

## Discussions

Improving therapeutic communication, as a standard ingredient of the bio-psycho-social (BPS) model of care

The most efficient way to implement and sustain a more positive attitude towards communication from physicians’ side seems to be the standard integration of communication training and practice into the BPS model of care. This model accommodates optimally to the needs and the perceptions of patients and has been consistently reported to lead to: 

- a significant improvement of patient’s satisfaction with care and of perceived health status [**24**-**26**];

- a genuine improvement of physician’s emotional competence and of diagnostic efficiency [**27**].

The bio-psycho-social model emphasizes the role of several components of communication, which illustrate essentially the so-called “patient-centered method” [**20**, **28**]:

- *exploring the disease and patient’s medical history:* doctors should evaluate the disease as a double process, involving objective and subjective changes. To get expertise regarding the first, medical training is often sufficient, however to acquire competence in the latter, it is necessary that the physician is equipped for understanding the unique patient perception and experience of the disease. In this sense, patient’s ideas regarding the disease, the impact of suffering over various functions and activities, the presence of hope are all elements which should be explored; 

- *understanding the patient:* implies that the doctors should consider the patient in his/her whole life context, in other words, an acknowledgement of multiple aspects, such as personality, personal history, microsocial environment (family) or macrosocial milieu (community). In order to perform this, doctors should make efforts for developing their assertive communicating abilities and the capacity of holistic approach;

- *finding a “shared starting point”*. This component was identified as being important in the foresight of positive results, which is why it is considered a solid element in patient-focused medicine. The techniques that can be used to realize it include identification (clarification) of the problem, establishment of treatment goals and identifying the roles which must be assumed by the patient and the doctor;

- *including the elements of prevention and promotion of health in the consultation:* each doctor-patient contact can be an opportunity to prevent and promote health. This can aim concrete activities (such as immunization), but also more abstract points (such as boosting self-confidence, formulation of positive suggestions or assuming healthy practices);

- *cultivating the solidity of the doctor-patient relationship:* this aspect is grounded on the development of compassion, empathy, trust, spirituality and sharing of initiative, power and responsibility; 

- *cultivating realistic therapeutic goals:* involves a degree of rationality for both actors involved in the therapeutic relationship. In what the physician is concerned, in order to avoid the burnout syndrome and its consequences, rationality implies acknowledging that, in numerous aspects of medical practice, team work is better for obtaining a better management of the disease and possibly a better prognosis for the patient.

**Monitoring adherence and perceived quality of life, as indicators of a good therapeutic relationship**

Efficient therapeutic communication can be measured indirectly through its impact on a series of parameters, among which *adherence* and *quality of life.*

Enabling the patient with a partner role has been proven to have substantial positive effects on *adherence*, via positive transference and increasing trust. Di Matteo et al. [**29**] identified, for example, a positive correlation between adherence and physician’s ability to identify various emotional states of the patient, starting from his/her nonverbal behavior. Other authors [**30**, **31**] emphasize the direct benefit on adherence brought by the precise and well-adapted explanations of the doctor. Trained communication skills of the doctor tend to influence positively the adherence of patients [**32**]. Nuanced and personalized doctor-patient communication can have also ethical implications, which are profoundly positive, in the sense that they offer a genuine framework for respecting an extensive list of patient’s rights, beginning with the right to be informed.

In what concerns *quality of life*, although this is a concept influenced by a large number of individual, psychosocial and environmental factors, a good doctor-patient relationship, built on the base of an efficient communication, proved to bring a significant plus even in chronic, poorly tolerated medical conditions. In these diseases, among the improvement of daily functioning, a good doctor-patient relationship has a real efficacy in improving the ability to tolerate pain and even to decrease the evolution of the disease [**33**, **34**]. A key element seems to be the patient’s sense of control (*mastery*), which is improved by a good chemistry with the doctor. Moreover, psychiatric comorbidity of these patients is typically low [**10**, **19**, **35**, **36**], as well as the costs of hospitalization and the necessity of medication [**37**]. 

For all reasons mentioned above, investing in improving the quality of doctor-patient communication increasingly becomes a priority of guidelines and protocols for the management of many diseases, be them strictly somatic, psychological or psychosomatic. The efficacy of improving communication skills tends to be remanent and cost-effective, a fact with powerful individual and social implications. 

## References

[1] Degner LF, Kristjanson LJ, Bowman D, Sloan JA, Carriere KC, O'Neil J, Bilodeau B, Watson P, Mueller B (1997). Information needs and decisional preferences in women with breast cancer. JAMA: The Journal of the American Medical Association.

[2] Gattellari M, Butow PN, Tattersall MHN (2001). Sharing decisions in cancer care. Social Science & Medicine.

[3] *Schmid Mast M, Klöckner C, Hall JA (2010). Gender, power, and non-verbal communication, in Kissane DW, Bultz BD, Butow PM, Finlay IG (Eds.). Handbook of communication in oncology and palliative care.

[4] *Bredart A, Bouleuc C, Dolbeault S (2005). Doctor-patient communication and satisfaction with care in oncology. Current Opinion In Oncology.

[5] Arora N (2003). Interacting with cancer patients: the significance of physicians’ communication behavior. Social Science \& Medicine.

[6] Lee S, Back A, Block S, Stewart S (2002). Enhancing physician-patient communication. ASH Education Program Book.

[7] Hall J, Roter D, Rand C (1981). Communication of affect between patient and physician. Journal of Health and Social Behavior.

[8] DiMatteo M (1998). The role of the physician in the emerging health care environment. Western Journal of Medicine.

[9] The A, Hak T, Koeter G, van der Wal G (2001). Collusion in doctor-patient communication about imminent death: an ethnographic study. Western Journal of Medicine.

[10] *Maguire P, Pitceathly C (2002). Key communication skills and how to acquire them. BMJ: British Medical Journal.

[11] *Fishbein M, Ajzen I (1975). Belief, Attitude, Intention and Behavior.

[12] *Fishbein M (1979). A theory of reasoned action: Some applications and implications, in Howe H, Page M. (Eds.). Nebraska Symposium on Motivation (Vol. 27).

[13] Popp I (2013). Personality disorders and their implications for the doctor-patient relationship. in Popa-Velea, O. Behavioral Sciences in Medicine.

[14] Cioca I, Popa-Velea O (2013). Verbal and nonverbal communication. in Popa-Velea, O. Behavioral Sciences in Medicine.

[15] *Armstrong TL, Swartzman LC (2001). Models and expectations for the health care provider-client/patient interaction in Kazarian SS, Evans DR. (Eds.). Handbook of Cultural Health Psychology.

[16] *Triandis HC, Trafimow D (2001). Culture and its implications for intergroup behavior, in Brown R, Gaertner SL. (Eds.). Blackwell Handbook of Social Psychology: intergroup processes.

[17] Popa-Velea O, Ţambu A, Diaconescu L (2013). Anamnesis styles, in Popa-Velea. O. Behavioral Sciences in Medicine.

[18] *Fiscella K, Meldrum S, Franks P, Shields CG, Duberstein P, McDaniel SH, Epstein RM (2004). Patient trust: Is it related to patient-centered behavior of primary care physicians?. Medical Care.

[19] *Stewart M, Brown JB, Donner A, McWhinney IR, Oates J, Weston WW, Jordan J (2000). The impact of patient-centered care on outcomes. Journal of Family Practice.

[20] *Margalit APA, Glick SM, Benbassat J, Cohen A (2004). Effect of a biopsychosocial approach on patient satisfaction and patterns of care. Journal of General Internal Medicine.

[21] *Alberti R, Emmons M (1995). Your perfect right: a guide to assertive living.

[22] *Hellstroem O (1998). Dialogue medicine: a health-liberating attitude in general practice. Patient Education & Counseling.

[23] *Roter DL, Hall JA (1992). Doctors talking to patients / Patients talking to doctors: improving communication in medical visits.

[24] Kinnersley P, Stott N, Peters T, Harvey I (1999). The patient-centredness of consultations and outcome in primary care. British Journal of General Practice.

[25] Safran D, Taira D, Rogers W, Kosinski M, Ware J, Tarlov A (1998). Linking primary care performance to outcomes of care. The Journal of Family Practice.

[26] Levinson W, Roter DL, Mullooly JP, Dull VT, Frankel RM (1997). Physician-patient communication. The relationship with malpractice claims among primary care physicians and surgeons. Journal of the American Medical Association.

[27] *Adler MH (2002). The sociophyiology of caring in the doctor-patient relationship. Journal of General Internal Medicine.

[28] *Stewart M, Gilbert BW (2005). Reflections on the doctor–patient relationship: from evidence and experience. British Journal of General Practice.

[29] DiMatteo MR, Hays RD, Prince LM (1986). Relationships of physicians' nonverbal communication skill to patient satisfaction, appointment noncompliance, and physician workload. Health Psychology.

[30] Savage R, Armstrong D (1990). Effect of a general practitioner's consulting style on patients' satisfaction: a controlled study. BMJ: British Medical Journal.

[31] Hall JA, Roter DL, Katz NR (1988). Meta-analysis of correlates of provider behavior in medical encounters. Medical Care.

[32] Diaconescu L, Popa-Velea O (2006). [Relaţia medic-pacient] Doctor-patient relationship, in Popa-Velea O, Diaconescu L, Necula Cioca I. [Psihologie Medicală. Baze teoretice şi aplicaţii clinice pentru medici şi psihologi] Medical Psychology. Theoretical bases and clinical applications for doctors and psychologists.

[33] Roter DL (1983). Physician / patient communication: transmission of information and patient effects. Maryland State Medical Journa.

[34] Greenfield S, Kaplan S, Ware JE Jr (1985). Expanding patient involvement in care. Effects on patient outcomes. Annals of Internal Medicine.

[35] Henrdon J, Pollick K (2002). Continuing concerns, new challenges, and next steps in physician-patient communication. Journal of Bone and Joint Surgery.

[36] Roter DL, Hall JA, Aoki Y (2002). Physician gender effects in medical communication: a meta-analytic review. JAMA.

[37] Little P, Everitt H, Williamson I, Warner G, Moore M, Gould C (2001). Observational study of effect of patient centredness and positive approach on outcomes of general practice consultations. BMJ: British Medical Journal.

